# Competing Endogenous RNA and Coexpression Network Analysis for Identification of Potential Biomarkers and Therapeutics in association with Metastasis Risk and Progression of Prostate Cancer

**DOI:** 10.1155/2019/8265958

**Published:** 2019-08-05

**Authors:** Xiaocong Pang, Ying Zhao, Jinhua Wang, Wan Li, Qian Xiang, Zhuo Zhang, Shiliang Wu, Ailin Liu, Guanhua Du, Yimin Cui

**Affiliations:** ^1^Department of Pharmacy, Peking University First Hospital, Xicheng District, Beijing 100034, China; ^2^Institute of Materia Medica, Chinese Academy of Medical Sciences and Peking Union Medical College, Beijing 100050, China; ^3^Department of Urology, Peking University First Hospital, Xicheng District, Beijing 100034, China

## Abstract

Prostate cancer (PCa) is the most frequently diagnosed malignant neoplasm in men. Despite the high incidence, the underlying pathogenic mechanisms of PCa are still largely unknown, which limits the therapeutic options and leads to poor prognosis. Herein, based on the expression profiles from The Cancer Genome Atlas (TCGA) database, we investigated the interactions between long noncoding RNA (lncRNA) and mRNA by constructing a competing endogenous RNA network. Several competing endogenous RNAs could participate in the tumorigenesis of PCa. Six lncRNA signatures were identified as potential candidates associated with stage progression by the Kolmogorov-Smirnov test. In addition, 32 signatures from the coexpression network had potential diagnostic value for PCa lymphatic metastasis using machine learning algorithms. By targeting the coexpression network, the antifungal compound econazole was screened out for PCa treatment. Econazole could induce growth restraint, arrest the cell cycle, lead to apoptosis, inhibit migration, invasion, and adhesion in PC3 and DU145 cell lines, and inhibit the growth of prostate xenografts in nude mice. This systematic characterization of lncRNAs, microRNAs, and mRNAs in the risk of metastasis and progression of PCa will aid in the identification of candidate prognostic biomarkers and potential therapeutic drugs.

## 1. Introduction

Prostate cancer (PCa) is the most frequently diagnosed malignant neoplasm and second leading cause of death in men worldwide [1]. Tumor metastasis is responsible for the majority of deaths [2]. Lymph node metastasis (LNM) is the most important risk factor for treatment in early-stage PCa [3]. However, the underlying pathogenic mechanisms of PCa are still largely unknown, which limits prognosis and therapy. The identification of new potential biomarkers and therapeutic targets for the progression of PCa would help overcome these serious clinical challenges and improve alternative therapies.

Noncoding RNAs (ncRNAs) have become recognized as important molecules in many types of cancer. They are potential biomarkers and can reveal uncharacterized aspects of tumor etiology. Accumulated evidence indicates that long ncRNAs (lncRNAs) participate in cellular processes of PCa. Relevant lncRNAs include PCAT1, PCAT5, PCA3, PCGEM1, MALAT1, PRNCR1, CTBP1-AS, TRPM2, and SCHLAP1 [4–11]. However, few PCa-related or specific lncRNAs have been well characterized. PCAT1 (prostate cancer-associated transcript 1) is a prostate-specific transcriptional repressor of cell proliferation and a target of the polycomb repressive complex 2 (PRC2) [4]. PCA3 (prostate cancer-associated 3) is a prostate-specific lncRNA that is involved in the control of cell survival, via the modulation of androgen receptor (AR) signaling [9]. MALAT1 (metastasis-associated lung adenocarcinoma transcript 1) is overexpressed during the progression of PCa and plays a vital role in enhancing zeste homolog 2- (EZH2-) promoted migration and invasion in castration-resistant PCa cell lines [10]. lncRNAs can also act as competing endogenous RNAs (ceRNAs) for competing microRNA (miRNA) involved in tumor etiology [12]. However, the mechanism of ceRNAs in PCa has not been extensively studied [13].

With the development of high-throughput sequencing technology, multiomics analyses have been successful in generating copious sequencing data and databases. This has created the need for informatics approaches to analyze and interpret relevant multiomics data. The approaches include support vector machine and random forest [14]. The machine learning approach has been rarely used to predict prostate metastasis risk and progression, and the prevalent methods of identifying molecular biomarkers need to be improved. Advances in genomics and bioinformatics have made drug repositioning a powerful alternative strategy to discover and develop novel anticancer drug candidates from existing drugs *in silico* [15]. For example, niclosamide, a Food and Drug Administration- (FDA-) approved antihelminthic drug, can inhibit the expression of AR variants and overcome enzalutamide resistance in CRPC [16].

**Figure 1:**
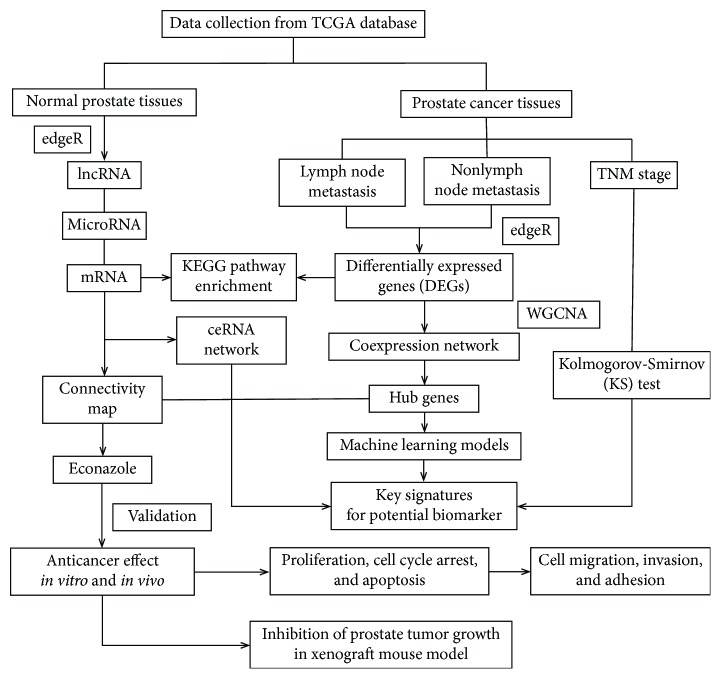
The workflow for systematic analysis of the characterization of lncRNAs, miRNAs, and mRNAs in the risk of metastasis and progression of prostate cancer, identifying new potential biomarkers, and therapeutic compound based on drug repositioning strategy.

The present study investigates the altered regulatory relationships of lncRNAs, miRNAs, and mRNAs between PCa patients and normal samples by constructing a competing endogenous RNA (ceRNA) network. This strategy identified several novel lncRNAs as functional ceRNAs with key roles in the pathogenesis of PCa and identified six stage-associated lncRNA signatures for PCa progression. Then, based on machine learning algorithms and hub genes from the coexpression network established by weighted gene coexpression network analysis (WGCNA) [17], we constructed four classifiers for the prediction of PCa prostate lymphatic metastasis: logistic regression (LR) [18], random forest (RF) [19], *k* nearest neighbors (kNN) [20], and naive Bayesian (NB) [21]. By targeting the coexpression network within PCa metastasis, the antifungal compound econazole was identified as a repositioning candidate on the basis of the putative pattern of action in the connectivity map (CMAP) [22]. Finally, we were the first to investigate the effects of econazole on the apoptosis, cell cycle, migration, invasion, and cell adhesion of PC3 and DU145 cell lines *in vitro* and its therapeutic effects against PCa xenografts in nude mice. The workflow was summarized in Figure 1, which made a substantial strategy for understanding the roles of lncRNAs, miRNAs, and mRNAs in tumorigenesis and progression of PCa and contributed to the discovery of new potential biomarkers and therapeutic drugs.

## 2. Materials and Methods

### 2.1. Data Collection and Processing

The mRNA, lncRNA, and miRNA transcriptome profiling of 489 PCa patients and 52 normal samples (adjacent to tumors) was retrieved from The Cancer Genome Atlas (TCGA) data portal (version October, 2017). The expression data of miRNA (miRNA-seq), lncRNA, and mRNA (RNAseqV2) were obtained using the Illumina HiSeq platform considering level 3. According to the TNM stage, 75 samples were identified as LNM and 292 samples were obtained from PCa tissue with non-LNM. There were three stages of PCa diagnosis recorded in the TCGA database: stage II (*n* = 116), stage III (*n* = 238), and stage IV (*n* = 8). In addition, mRNA and lncRNA annotation and extraction were performed using the ENSEMBL genome database. After background correction and quantile normalization, 17866 mRNAs, 7997 lncRNAs, and 696 microRNAs were further analyzed. In this paper, we mainly used the program code written in Perl and R language to analyze and deal with RNA data. Differential expression analysis of mRNAs, lncRNAs, and miRNAs was conducted using edgeR with a false discovery rate (FDR) threshold < 0.05 and an absolute value of log_2_FC > 1.5 to judge the significance of the gene expression differences.

### 2.2. Construction of the ceRNA Network

We integrated the expression data of lncRNAs, miRNAs, and mRNAs with its related database information to construct the ceRNA network. To further improve the ceRNA network reliability, we retained lncRNAs, miRNAs, and mRNAs included in different expression of RNAs between tumor tissues and normal tissues. The miRNA-lncRNA interactions were predicted according to the miRcode database (http://www.mircode.org/), which is a comprehensive searchable map of putative miRNA target sites across the complete GENCODE-annotated transcriptome, including >10000 lncRNA genes [23]. Next, to retrieve miRNA-targeted mRNAs, we integrated the information from miRDB, TargetScan, and miRTarBase databases. miRDB (http://mirdb.org) is an online database for miRNA target prediction and functional annotations that includes 2.1 million predicted gene targets regulated by 6709 miRNAs, which is based on high-throughput studies, and also combines computational analyses and literature mining [24]. TargetScan (http://www.targets.org) predicts miRNA target interactions by seeking existing and conserved 8mer, 7mer, and 6mer sites that match the seed region of each miRNA [25]. miRTarBase (http://miRTarBase.mbc.nctu.edu.tw/) provides information about experimentally validated miRNA target interactions. The intersection of prediction results from the three databases was utilized to establish miRNA-mRNA interactions [26]. Cytoscape 3.6.0 software was used to visualize the ceRNA network.

According to the ceRNA hypothesis, when the lncRNA and mRNA competed for the same miRNA, the lncRNA-miRNA-mRNA triplet was regarded as a potential ceRNA triplet. We next calculated the mutual information (MI) and conditional mutual information (CMI) to identify ceRNA triplets [27]. The quantities and their associated statistical significance were computed based on lncRNA, miRNA, and mRNA expression profiles. The possibility of lncRNA and mRNA interaction was computed by the following equation [28]:

(1) $$\begin{array}{c} \Delta I=I\left[\text{miRNA};\text{mRNA}\;|\;\text{lncRNA}\right]-I\left[\text{miRNA};\text{mRNA}\right]. \end{array}$$

*I*[miRNA; mRNA] represents the MI between miRNA and mRNA, which shows the relationships between miRNA and mRNA; *I*[miRNA; mRNA | lncRNA] represents the CMI of miRNA and mRNA after adding the variable lncRNA; and Δ*I* denotes the significance of lncRNA acting as a miRNA sponge. Mimic Δ*I* values were calculated by random permutation of the lncRNA expression across samples 500 times. By comparing the real and mimic Δ*I* values, a *P* value was calculated.

### 2.3. Pathway Enrichment and Progression of PCa Analysis

To study pathological mechanisms of PCa and lymphatic metastasis, the differentially expressed genes (DEGs) between tumor tissues and normal tissues and between LNM and non-LNM tissue from PCa were conducted Kyoto Encyclopedia of Genes and Genomes (KEGG) pathway enrichment analysis by the clusterProfiler R package. The analysis and visualization modules were obtained from the Bioconductor project. The Kolmogorov-Smirnov (KS) test is a robust nonparametric testing method that can be applied for comparison of multiple groups without a known distribution pattern [29]. The KS test was performed using the R package to analyze the pattern of variability among stages II, III, and IV and to identify potential biomarkers to predict the progression of PCa.

### 2.4. Weighted Gene Coexpression Network Analysis (WGCNA)

The coexpression network was constructed by the WGCNA R package by calculating robust correlations between all genes across all relevant LNM and non-LNM samples. Based on scale-free topology criterion, the correlation adjacency matrix was increased to the power of *β* = 12 to amplify the strong connections between genes and penalizing the weaker connections. The first principal component is considered as the module eigengene (ME), which denotes the highest percent of variance for all the genes in a module. Module membership (kME) determines the correlations between each gene and each ME [30, 31]. The within-module connectivity for each gene is evaluated by computing the connectivity of that gene with each other gene set in that module. The hub genes with significant correlations with MEs and high within-module connectivity were obtained for further study.

### 2.5. Establishment of Lymphatic Metastasis Prediction Models

#### 2.5.1. Signature Screening

The hub genes from the coexpression network served as the descriptors for building the lymphatic metastasis prediction models. Pearson correlation analysis was used to eliminate low correlation by calculating the correlation coefficient between the metastasis state (nonlymphatic metastasis and lymphatic metastasis samples are marked “1” and “-1,” respectively) and descriptors. The descriptor whose correlation coefficient with the metastasis state was <0.1 was deleted. After filtration, the selected descriptors served as signatures to establish the prediction models.

#### 2.5.2. Establishment and Performance of Models

To discriminate LNM from non-LNM for PCa, LR, RF, kNN, and NB machine learning classifiers were used. LR provides a deterministic model yielding weighting factors for each contributing variable. However, unlike linear discriminant analysis, it does not require the independent variables to be normally distributed, linearly related, or of equal variance within each group [18, 32]. RF utilizes an ensemble of unpruned decision trees [19], each of which is constructed on a bootstrap sample of the training data using a randomly selected subset of variables [33]. The input data were divided into two subsets based on a particular molecular descriptor and corresponding splitting value at each node of the decision tree. When there were no more significant nodes, the splitting process was finished [34]. In data segmentation clustering methods, the most widely used and well-known method is kNN. The kNN algorithm could classify objects according to the closest examples in the feature space [35]. An object is classified by a majority vote of its neighbors, with the object being assigned to the class most common among its *k* nearest neighbors. NB is widely used as a probabilistic classification model [21]. The Bayesian algorithm computes the posterior probability directly based on the kernel function of the following equation [36]:

(2) $$\begin{array}{c} P\left(A\;|\;B\right)=P\left(B\;|\;A\right)*\frac{P\left(A\right)}{P\left(B\right)}. \end{array}$$

*P*(*A* | *B*) represents the probability of *A* assuming that *B* is true, which is the posterior probability of the model; *P*(*A*) is the prior probability and refers to the probability in the hypothesis space; and *P*(*B* | *A*) is the likelihood of the model.

LR, RF, kNN, and NB were performed in Orange canvas 3.4.1. 75 LNM and 292 non-LNM samples were used as the training set for model building. The performance of the classification algorithms was estimated by 5-fold cross-validation, random sampling (*n* = 10), and leave-one-out validation.

### 2.6. Screening of Drug-Like Small Molecules

CMAP (http://www.connectivitymap.org/cmap/) [37] was used to collect the genome-wide transcriptional expression data from cultured human cells treated with bioactive small molecules and simple pattern-matching algorithms that together facilitate the discovery of functional connections between drugs, genes, and diseases. The hub genes for lymphatic metastasis and DEGs in PCa samples compared with normal tissues were divided into two groups of upregulated and downregulated genes and uploaded as an input set in CMAP. By comparing the expression pattern similarities of the differential genes and genes perturbed by compounds in CMAP, small molecules involved in the lymphatic metastasis of PCa were identified.

### 2.7. Validation of Drug Repositioning in PCa

#### 2.7.1. Reagents and Antibodies

Econazole (CAS no. 24169-02-6) was purchased from MedChemExpress Company (Beijing, China). Fetal bovine serum (FBS), RPMI 1640 medium, and penicillin/streptomycin were obtained from Gibco/Life Technologies (Gaithersburg, MD, USA). The CCK-8 Assay Kit was purchased from Japan Tongren Chemical Co. (Kyushu Island, Japan). The EdU Cell Proliferation Assay Kit was purchased from Beijing Solaibao Technology Co. Ltd. (Beijing, China). The JC-1 Mitochondrial Membrane Potential Detection Kit and Cell Cycle Assay Kit were purchased from Beyotime Biotechnology (Jiangsu, China). The Annexin V-fluorescein/propidium iodide (FITC/PI) Apoptosis Detection Kit was obtained from Absin Bioscience Inc. (Shanghai, China). Matrigel matrix and Transwell chambers were purchased from Corning Inc. (Corning, NY, USA). Bovine serum albumin (BSA), dimethyl sulfoxide, and ribonuclease (RNase A) were purchased from Sigma-Aldrich (St. Louis, MO, USA). Pierce™ BCA, protein assay kit, protease inhibitor cocktail, and SuperSignal West Pico Chemiluminescent Substrate detection kit were purchased from Thermo Fisher Scientific (Waltham, MA, USA). Polyvinylidene difluoride membranes were purchased from Millipore (Billerica, MA, USA). RIPA lysis buffer and antibodies to phosphor-Rb (P-Rb), CyclinE, p53 upregulated modulator of apoptosis (PUMA), myeloid leukemia cell differentiation protein-1 (MCL-1), B-cell lymphoma 2 (BCL-2), poly (ADP-ribose), PARP, caspase 9, and glyceraldehyde-3-phosphate dehydrogenase (GAPDH) were obtained from Cell Signaling Technology Inc. (Beverly, MA, USA).

#### 2.7.2. Cell Culture and Proliferation Assay

PC3 and DU145 cells were purchased from the Institute of Basic Medical Sciences, Chinese Academy of Medical Sciences. The cells were cultured in RPMI 1640 medium supplemented with 10% FBS in a humidified atmosphere with 37°C and 5% CO_2_. The PC3 and DU145 cells (4 × 10^4^ cells/mL) were incubated with different concentrations of geldanamycin, 6-bromoindirubin-3′-oxime (BIO), and rilmenidine for 72 h. The concentration of geldanamycin was 0, 0.15, 0.46, 1.37, 4.12, 12.35, 37.04, 111.11, 333.33, or 1000 nM, and the concentrations of BIO and rilmenidine ranged from 0 to ~20 *μ*M and 0 to ~80 *μ*M, respectively, by double dilution method. The PC3 and DU145 cells (4 × 10^4^ cells/mL) were incubated with 0, 1.25, 2.5, 5, 10, 20, 40, 60, or 80 *μ*M econazole in 96-well plates for 12, 24, 48, and 72 h. Viable cells were measured by the CCK-8 assay. The absorbance value of each well was determined using a SpectraMax M5 ELISA microplate reader (Molecular Devices, Sunnyvale, CA, USA). Based on the specific reaction of Apollo® fluorescent dye and EdU, the replication activity of DNA could be directly and accurately detected, which reflected the effect of econazole on cell proliferation. Transfected cells (6 × 10^4^ cells/mL) were plated into 96-well plates with various concentrations of econazole (0, 5, 10, 20, and 40 *μ*M) for 24 h and 48 h. The samples were examined using the protocol of Cell-Light™ EdU Apollo® 488 *in vitro* Imaging Kit. The fluorescence intensity was detected and analyzed with a Cellomics ArrayScan VTI HCS Reader (Cellomics Inc., Pittsburgh, PA, USA) with the Morphology Explorer BioApplication software. The mean fluorescence intensity was assessed based on the EdU fluorescence, and the ratio of the mean fluorescence intensity of Edu that decreased following treatment with econazole was computed with the value of the control group designated as 100%.

#### 2.7.3. Cell Cycle Arrest and Apoptosis

PC3 and DU145 cells in the exponential phase of growth were digested into single cell suspension, and 2 mL was added to 6-well plates at a density of 1 × 10^5^ cells/mL. After the cells were completely attached, the serum-free medium was cultured for 18-24 h. After treatment with different concentrations of econazole for 48 h, the cells of the six-well plates were separately collected, washed once with precooled PBS, resuspended in 70% ethanol precooled at -40°C, and fixed at 4°C overnight. The cells were centrifuged at 1500 rpm for 5 min, and the supernatant was discarded. After washing twice, 200 *μ*L of PI staining solution was added and staining proceeded at room temperature for 30 min in the dark. The cells were filtrated through 400-mesh nylon mesh prior to detection of the cell cycle. For the analysis of apoptosis, PC3 and DU145 cells were seeded at a density of 5 × 10^5^ cells/mL. The Annexin V-FITC/PI Apoptosis Detection Kit was used to stain the cells following the manufacturer's instructions. Cell cycle and apoptosis analyses were performed using Flow Draw 10.0 software (https://www.draw.io). To further investigate the mechanism of econazole-induced cell cycle arrest and apoptosis in PCa cells, we examined the expression of cell cycle- and apoptosis-related proteins by western blotting. The targets were P-Rb, CyclinE, PUMA, P53, Bcl-2, Mcl-1, PARP, and cleaved PARP.

#### 2.7.4. Determination of Mitochondrial Membrane Potential (MMP)

JC-1 is a lipophilic cationic dye that accumulates in mitochondria depending on the MMP. Increasing MMP results in progressive accumulation of JC-1 in the mitochondria. PC3 and DU145 cells were cultured in 6-well plates overnight. The cells were treated with 0, 5, 10, 20, and 40 *μ*M econazole for 48 h. The cells were harvested, washed with PBS, and resuspended in PBS. JC-1 was then added to each sample. The samples were incubated in the dark for 30 min. Then, the cell pellets were obtained by centrifugation at 1000 rpm for 5 min and the cells were washed with PBS, resuspended in PBS, filtered, and examined using flow cytometry.

#### 2.7.5. Cell Migration and Invasion

PC3 and DU145 cells (5 × 10^4^) cultured with 0, 5, 10, 20, and 40 *μ*M econazole were suspended in 200 *μ*L serum-free medium and added to the upper chambers of Transwell devices. The lower chamber of each device contained 600 *μ*L of 10% FBS RPMI 1640 as a chemoattractant. After incubation for 12-18 h, cells that had not migrated to the lower chamber were carefully removed from the membrane exposed to the upper chamber using a cotton swab. The cells that had invaded to the lower chamber in response to the chemoattractant were fixed with 4% formaldehyde and stained using crystal violet for 10 min. A minimum of five separate fields was examined by fluorescence microscopy.

#### 2.7.6. Cell Adhesion Assay

Ninety-six-well culture plates were coated with 50 *μ*L of fibronectin. Three wells were coated with 1% BSA solution as a minimal adhesion control. The plate was placed at 4°C overnight. Cells were harvested and seeded at 2 × 10^5^ cells/mL and treated with 0, 5, 10, 20, and 40 *μ*M econazole in wells of precoated 96-well plates. After 2 h of incubation at 37°C, adherent cells were fixed with 4% paraformaldehyde for 30 min, washed with PBS, and stained with crystal violet overnight. The optical density value at 590 nm was determined using an ELISA microplate reader.

#### 2.7.7. Xenograft Experiment

BALB/c nude mice were obtained from Beijing Vital River Laboratory Animal Technology and were maintained in pathogen-free conditions. The mice were randomly classified into four groups (*n* = 8 mice per group). A 100 *μ*L suspension of logarithmic growth-phase PC3 cells (1 × 10^7^ cells/mL) was inoculated subcutaneously in the left groin of each mouse.

The tumor-bearing nude mice were fed in the barrier system and maintained until the tumor volume, determined as described below, reached 50 mm^3^. The mice were randomly allocated to the vehicle control group or one of the three econazole groups (20, 40, or 80 mg/kg). Each dose of econazole was administered by intraperitoneal injection once a day for 21 days. The body weight of each mouse was measured every 4 days. The longest and shortest diameters (mm) of the subcutaneous tumor of each nude mouse were measured using a vernier caliper, and the tumor volume was calculated as 0.52 × longest diameter × vertical diameter [2]. The mice were sacrificed on day 21, and the tumors were harvested and weighed. Immunohistochemical staining expression of Ki67 protein was done to evaluate the effect of econazole on tumor proliferation. The experimental protocol was approved by the Ethics Committee of Peking Union Medical College (Beijing, China). This study was implemented in accordance with the recommendations in the Guide for the Care and Use of Laboratory Animals of the National Institutes of Health.

#### 2.7.8. Statistical Analyses

Data are presented as the mean ± standard deviation (SD) from at least three independent experiments. Statistical analyses and graph presentation were carried out using GraphPad Prism software version 6.0 (GraphPad Software, La Jolla, CA, USA). The significance of differences between groups was assessed by Student's *t*-test or one-way ANOVA. *P* values of statistical significance are represented as ^∗^ *P* < 0.05, ^∗∗^ *P* < 0.01, and ^∗∗∗^ *P* < 0.001.

## 3. Results

### 3.1. Functional Characterization of the ceRNA Network of PCa

**Figure 2:**
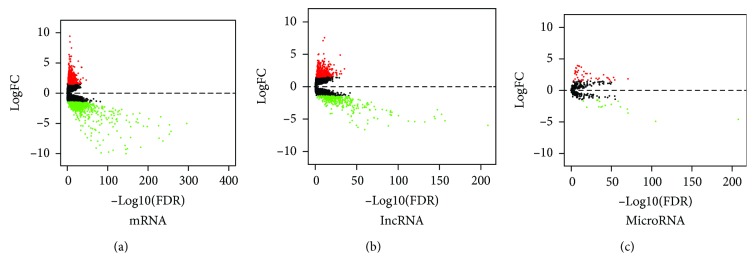
Volcano plots for mRNA (a), lncRNA (b), and miRNA (c). The red and green spots represent upregulated and downregulated differentially expressed genes (DEGs), respectively. logFC values are log_2_(FC).

**Figure 3:**
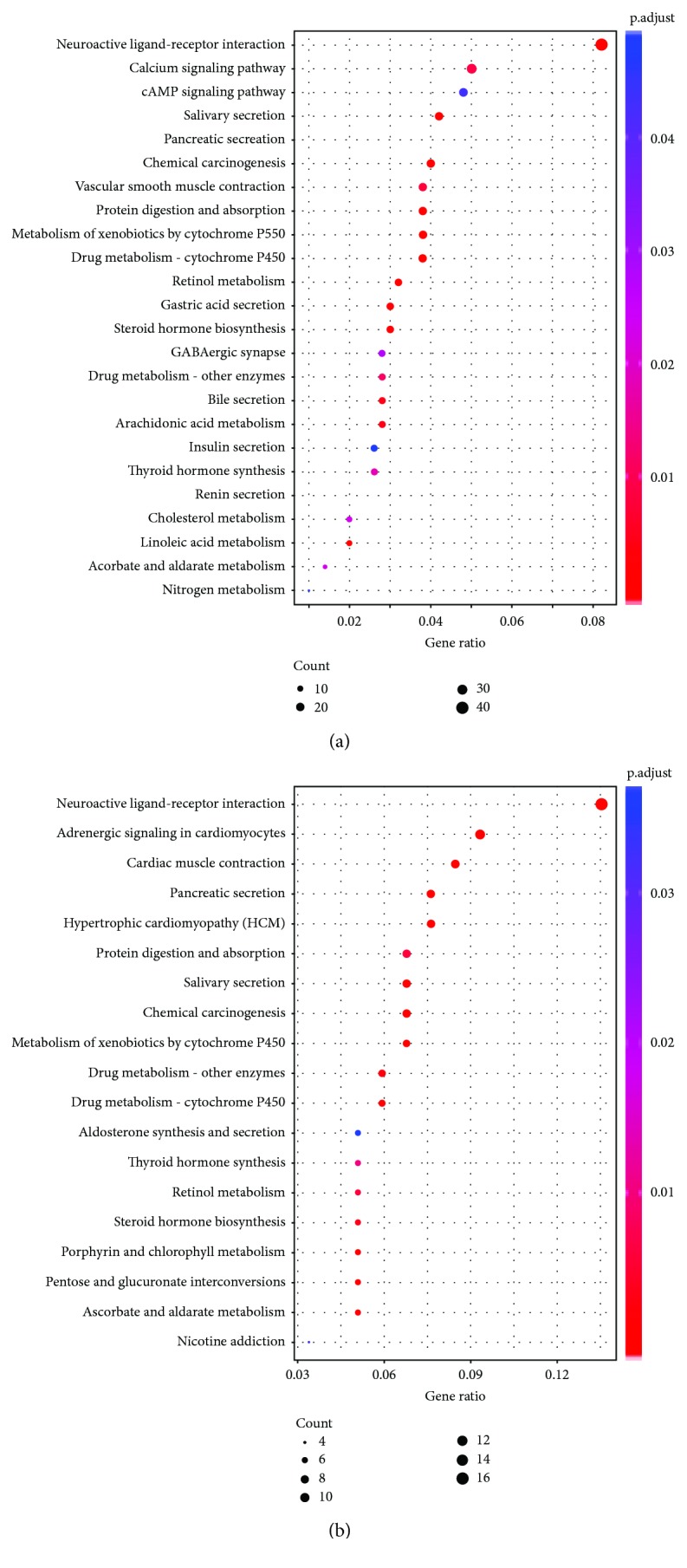
KEGG pathway enrichment for DEGs. (a) Enriched pathways for DEGs between PCa and normal samples. (b) Enrichment analysis after comparing DEGs of PCa lymphatic metastasis and nonmetastasis tissues.

This study investigated the expression levels of mRNA, lncRNA, and miRNA in tumor and normal tissues. Differentially expressed RNAs were filtered using the criteria of logFC = 1.5 and corrected *P* value (FDR) < 0.05 using the edgeR package. These criteria identified 660 upregulated and 739 downregulated mRNAs, 441 upregulated and 361 downregulated lncRNAs, and 43 upregulated and 16 downregulated miRNAs in tumors compared to normal tissues. Volcano plots were generated (Figure 2). KEGG pathway enrichment was performed for differentially expressed genes (DEGs). Neuroactive ligand-receptor interaction was the most significantly enriched, with the calcium signaling pathway and metabolism-related pathway also being appreciably enriched (Figure 3(a)). Similar results were observed in PCa lymphatic metastasis-related pathway enrichment (Figure 3(b)). Neuroactive ligand-receptor interaction, especially the GABAergic system, has significant effects on the progression of PCa. The GABAergic system is enriched in neuroendocrine cell- (NE-) like cells [38] and contributes to PCa progression due to the secretion of neuropeptides [39].

**Figure 4:**
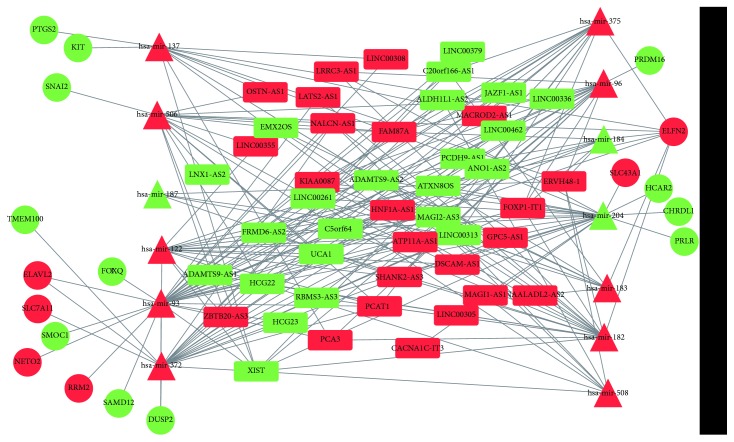
ceRNA network of PCa. The red and green parts represent upregulated and downregulated RNAs, respectively. Rectangle, circle, and triangle denote lncRNA, mRNA, and miRNA, respectively.

To clarify the roles of differentially expressed lncRNAs and the regulatory interactions of ceRNAs presented in normal and PCa tissues, we constructed the lncRNA-miRNA-mRNA network of PCa. We first used the miRcode database to retrieve the differentially expressed lncRNAs and the potentially related miRNAs. Then, the Perl program was used to extract 1643 pairs of interacting lncRNAs and miRNAs. After excluding the miRNAs that were not differentially expressed, an interaction of 182 pairs of lncRNAs (*n* = 46) and miRNAs (*n* = 13) was obtained. Next, the targeted mRNAs of these miRNAs were retrieved from the miRTarBase, miRDB, and TargetScan. A total of 18 differentially expressed mRNAs were common to the three databases. These were used to construct the ceRNA regulatory network of PCa by incorporating 46 lncRNAs, 18 mRNAs, and 13 miRNAs (Figure 4). The Δ*I* values for the ceRNA triplets were calculated. The permutation test was used to compute the *P* value of the triplets. Three triplets with *P* < 0.01 were identified: *XIST*-miR 372-*DUSP2*, *LINC00336*-miR 96-*PRDM16*, and *EMX2OS*-miR 508-*SNAI2*.

### 3.2. Prognostic lncRNA Biomarkers Associated with Progression of PCa

**Figure 5:**
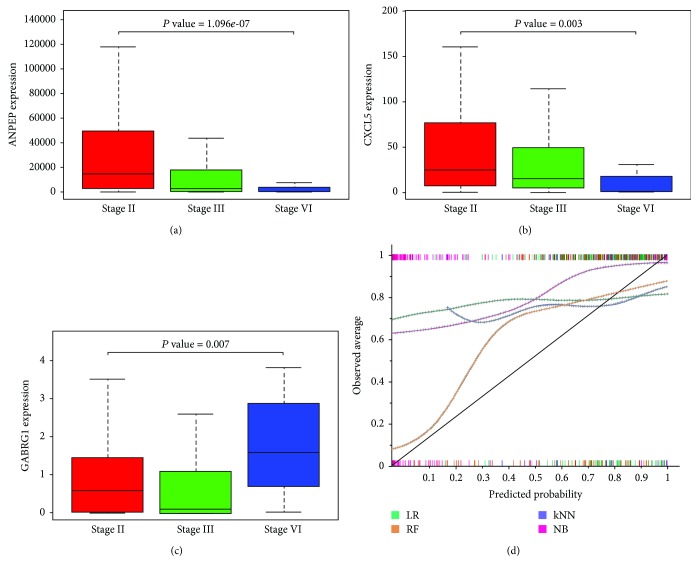
The prognostic values of mRNAs in PCa were evaluated by KS test to analyze the pattern of variability among stages II, III, and IV. *P* values of *ANPEP* (a), *CXCL5* (b), and GABRG1 (c) were lower than 0.01. The calibration plot of four lymphatic metastasis models (d). The relationship between measured values and predictive values can be observed visually by calibration plot. Among these classification models (LR, kNN, NB, and RF algorithms), the RF model best fits to the straight line, which suggested good predictive accuracy.

Accumulating evidence indicates that long lncRNA may affect the progression of PCa. We utilized the KS test to determine the prognostic value of lncRNAs in PCa. UCA1 and OSTN-AS1 were the key lncRNAs in the ceRNA network of PCa (both *P* < 0.05; Figure 4 and Figure S1). UCA1 was downregulated in PCa tissue from the ceRNA network, but expression gradually increased with the progression of PCa. More importantly, UCA1 was also upregulated in PCa lymphatic metastasis. Therefore, UCA1 was a highly sensitive signature for the prediction of the progression of PCa. In addition, SCHLAP1, LINC01141, CTD-2521M24.5, and RP11-245J24.1 were identified as being important indicators of the progression of PCa-related lncRNAs (all *P* < 0.01; Supplement figure). Three genes (ANPEP, CXCL5, and GABRG1) also displayed significant relationships with clinical stages (all *P* < 0.01; Figures 5(a)–5(c)).

### 3.3. Dysfunctional Coexpression Network Construction in PCa Lymphatic Metastasis

**Figure 6:**
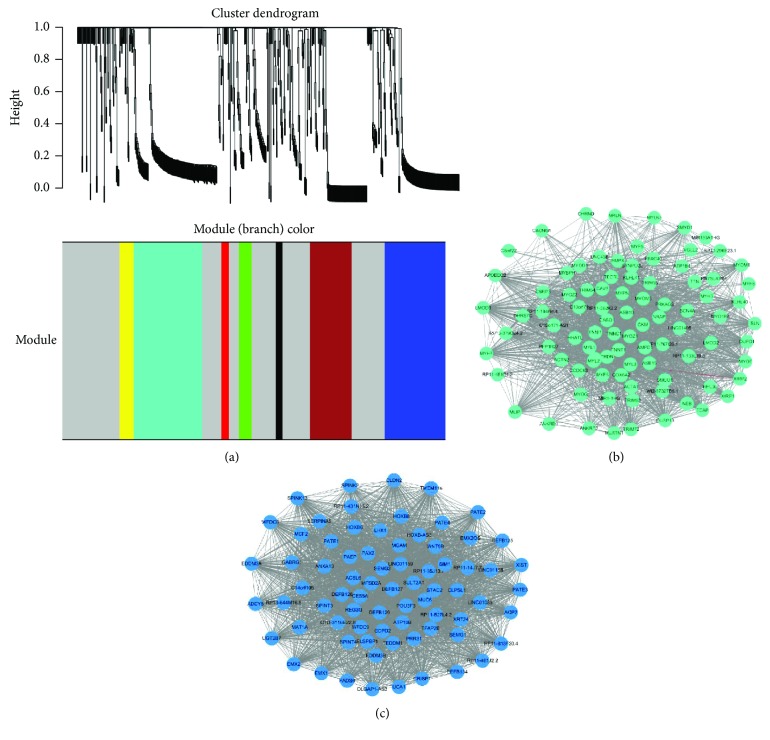
Dysfunctional coexpression network construction in PCa lymphatic metastasis. (a) Dendrograms produced by average linkage hierarchical clustering of genes. Modules are defined as clusters of densely interconnected genes. Hierarchical clustering of module eigengenes that summarize the modules found in the clustering analysis. Branches of the dendrogram group together eigengenes that are positively correlated. The extent of gene conservation in the datasets was represented by the same module colors. The top two enriched modules were presented as (b) turquoise and (c) blue. The genes of “highly connected gene” inside coexpression modules tend to be hub genes.

To understand the mechanism of LNM and prognostic signs for the dissemination of PCa, we compared the gene expression profiles of lymphatic metastasis samples (*n* = 292) and nonlymphatic metastasis samples (*n* = 75) in PCa. WGCNA was used utilizing edgeR to analyze the coexpression of the DEGs. The adjacency cutoff value of WGCNA was set to 0.8. Based on WGCNA convection, the top two enriched modules depicted in turquoise and blue displayed good distribution, as did brown modules (Figure 6). Hub genes were evaluated by computing the degree to determine the connectedness. The hub gene with a degree > 10 (226 nodes) was selected to establish the PCa lymphatic metastasis models.

### 3.4. Establishment and Evaluation of Lymphatic Metastasis Models

The 226 nodes from the coexpression network were used as primary signatures for lymphatic metastasis prediction. We calculated the Pearson correlation coefficient between the 226 signatures with the state of lymphatic metastasis. After screening, 32 significant signatures (*P* < 0.05) were used as the input variables to establish the lymphatic metastasis models. They included CLDN2, CRISP1, UCA1, PATE2, HOXB8, EMX2, SERPINA5, SPINT3, PAEP, ELSPBP1, AQP2, UGT2B7, EDDM3B, TEDDM1, GABRG1, XIST, EDDM3A, PATE1, WNT9B, POU3F3, HOXB6, GDPD2, PAX2, SULT2A1, ACSL6, EMX1, ANPEP, SCHLAP1, CXCL5, TFAP2B, SIM1, and MUC6. Among these signatures, GABRG1, ANPEP, SCHLAP1, and CXCL5 were highly associated with the clinical stage (*P* < 0.01; Supplement figure and Figure 5).

All the classification models (LR, kNN, NB, and RF algorithms) were built using Orange canvas 3.4.1. Subsequently, the 5-fold cross-validation and leave-one-out methods were adopted to evaluate the performance of the four classifiers. Additionally, the training sets randomly were divided into 10 training and test sets with a 3 : 2 proportion. The performance of all the single classifiers is summarized in Table 1. The RF model displayed the best performance with an area under the curve and precise value of 0.840 and 0.842, respectively, in the 5-fold cross-validation. The precise values for the leave-one-out and random sampling for RF were 0.840 and 0.844, respectively. In the calibration plot (Figure 5(d)), the RF model best fits to the straight line, which suggested good predictive accuracy.

### 3.5. Small Molecules Involved in PCa

Using the two up- and downregulated gene groups as the input set for the CMAP database, the top ten small molecules that were reversed with the dysregulation of PCa were determined (Table 2). Geldanamycin had the highest enrichment score in the lymphatic metastasis group. The values of IC_50_ of geldanamycin were detected as 16.42 nM and 27.40 nM in PC3 and DU145 cells, respectively.  Figure S2 A, C showed the dose response curve of geldanamycin. It was reported that geldanamycin is an effective inhibitor of hsp90 for breast cancer and PCa treatment [40, 41]. Geldanamycin also induces degradation of hypoxia-inducible factor 1 alpha protein via the proteasome pathway in PCa cells [42]. However, geldanamycin has not been used clinically because of the toxicity associated with its solubility.  Figure S2 B, D showed the dose response curve of BIO, and IC_50_ values in PC3 and DU145 cells were 0.50 and 1.35 *μ*M, respectively. BIO is a glycogen synthase kinase 3 (GSK-3) inhibitor and has neuroprotective and regenerative effects [43]. Zhang et al. found that BIO significantly improves the targeting of antisense oligonucleotides (ASOs) in both the cell cytoplasm and the nucleus [44]. Furthermore, BIO enhances ASO function and represses AR expression through the inhibition of the two main GSK-3 isoforms: GSK-3*α* and GSK-3*β* activity [44]. However, Kohler et al. suggested that low-dose BIO induced increased neovascularization, secondary to VEGF, a process that was accompanied by a partial dedifferentiation of endothelial cells via *β*-catenin [45]. Therefore, BIO has double sides due to the dose usage and BIO is a typical tool medicine of the GSK-3 inhibitor, which is not applied in clinics. The antitumor effects of rilmenidine on PC3 and DU145 cells were poor, and both IC_50_ values were more than 60 *μ*M. Econazole displayed the second highest enrichment score of -0.956 in lymphatic metastasis and the highest score of -0.972 in PCa samples. Econazole is used clinically as an antifungal drug with many different *in vitro* effects. It was reported that econazole inhibits phosphoinositide-3-kinase activity and promotes apoptosis in non-small-cell lung cancer cell lines, including H661 and A549, with an effect on intracellular Ca^2+^ concentrations and the proliferation of PC3 cells [46]. Econazole could stimulate endoplasmic reticulum Ca^2+^ release and hyperpolarize the mitochondrial membrane and cause a rapid increase in oxidative stress (OS) in leukemia cells [47]. However, there has not been a systematic evaluation of econazole on the apoptosis and metastasis in human PCa lines. Therefore, we investigated the effect of econazole on the induction of apoptosis in PCa cells, inhibition of metastasis in vitro, and the antitumor activity in vivo.

**Figure 7:**
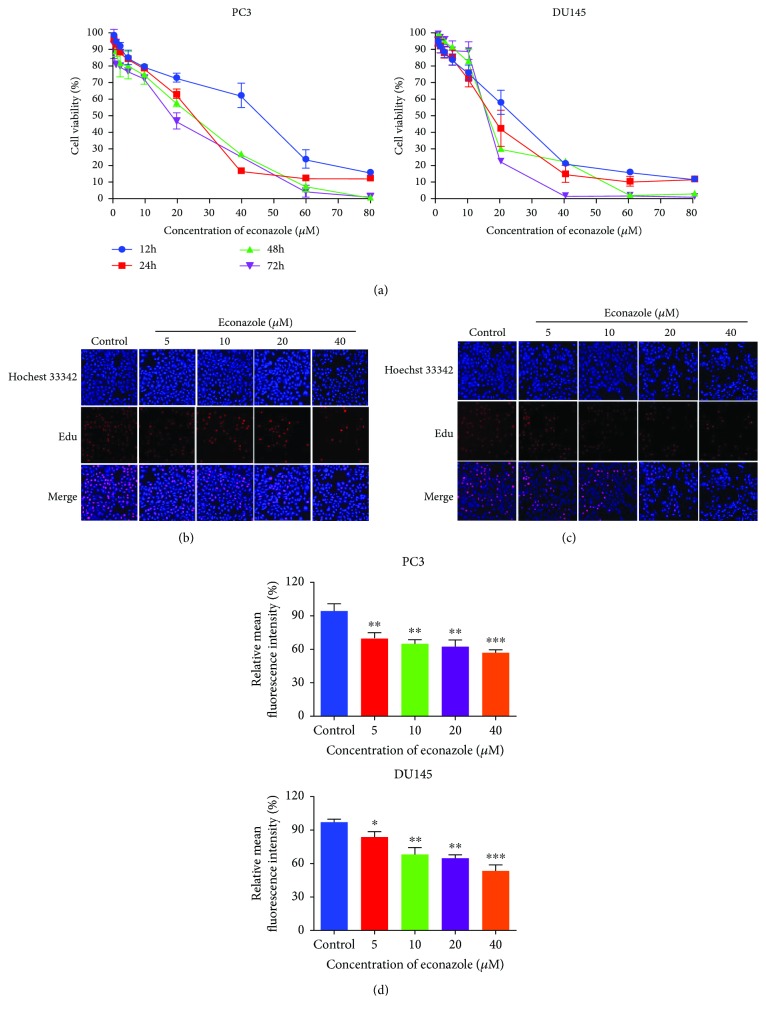
Econazole suppresses PC3 and DU145 cell proliferation. (a) PC3 and DU145 cells were treated with econazole for 12, 24, 48, and 72 h, and cell viability was assessed by the CCK-8 assay. (b, c) Immunofluorescence staining of PC3 and DU145 cells incubated with 0, 5, 10, 20, and 40 *μ*M econazole for 48 h was observed by fluorescence microscopy (magnification 100x). Proliferation cells were dyed with EdU in red, while whole cells were stained with Hoechst in blue. The EdU level was calculated by the high-content system based on fluorescence intensity. Statistical analysis of EdU assay was shown in (d) for PC3 and DU145 cells treated for 48 h. The data was reported by mean ± SD from three experiments. ^∗^ *P* < 0.05, ^∗∗^ *P* < 0.01, and ^∗∗∗^ *P* < 0.001.

Econazole inhibited PC3 and DU145 cell proliferation in time- and dose-dependent manners as determined in CCK-8 assays (Figure 7(a)). The EdU assay is an immunochemical detection method that measures nucleotide analogue incorporation into newly replicated DNA. The result of EdU assay was consistent with that from the CCK-8 assays. The relative mean fluorescence intensity was significantly lower in cells incubated with econazole, and it also showed time- and dose-dependent effects (Figures 7(b)–7(d)).

### 3.6. Validation of the Antitumor Activity of Econazole *In Vitro* and *In Vivo*

PC3 and DU145 cells were used to investigate the effects of econazole on the migration, invasion, and cell adhesion *in vitro*, and the inhibition of the growth of PCa xenografts in nude mice was further studied *in vivo*.

#### 3.6.1. Effect of Econazole on Cell Cycle and Apoptosis

**Figure 8:**
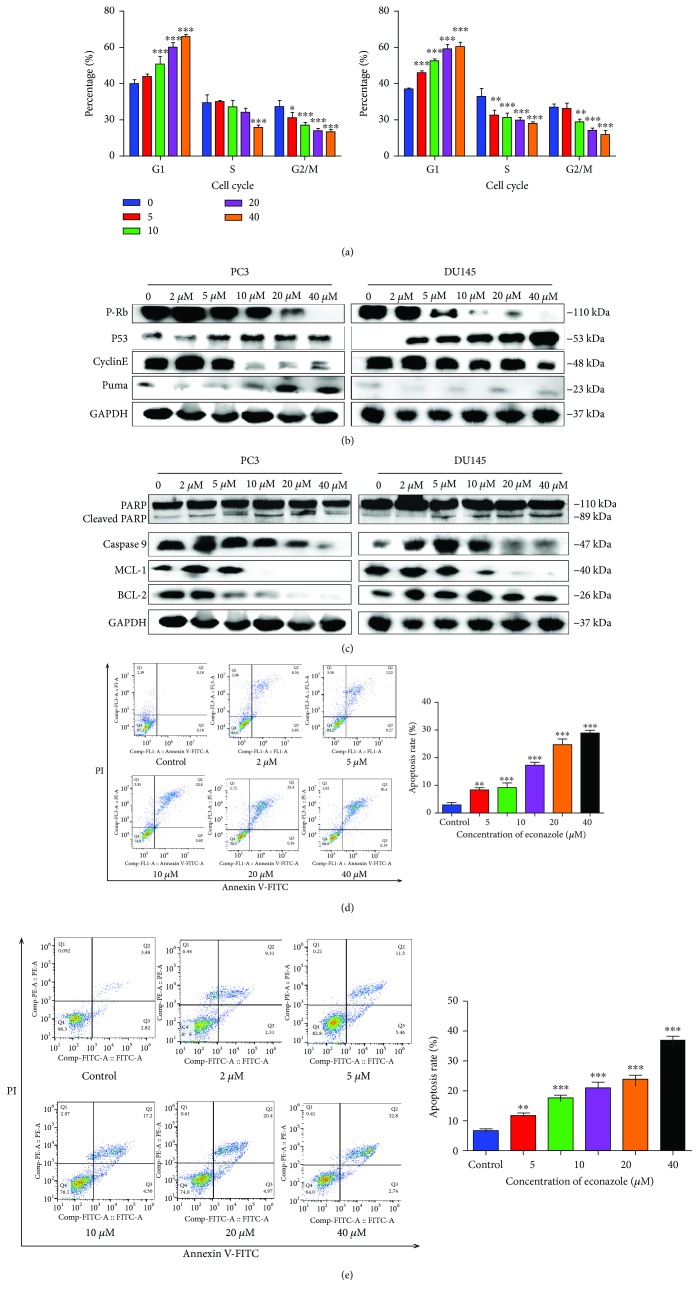
Effect of econazole on the cell cycle and apoptosis. (a) DU145 and PC3 cells were treated with 0, 5, 10, 20, and 40 *μ*M econazole for 48 h and then stained with PI for flow cytometry analysis. The percentages of PC3 cells at the G1 phase increased to 46.1%, 52.5%, 58.7%, 60.5%, respectively, compared with the control group (G1 = 36.8%). For DU145 cells treated with the same concentration of econazole, the percentages of cells in the G1 phase increased to 43.9%, 51.1%, 60.3%, and 65.9%. (b) Western blot analysis was performed to detect the expression levels of key proteins at the G1 phase. Econazole inhibited p-Rb and CyclinE expression and induced PUMA and P53 protein expression. (c) The expression of apoptosis-related proteins was detected by western blot. Econazole reduced the expression of Bcl-2, Mcl-1, and PARP and increased the expression of cleaved PARP. After being treated with various concentrations of econazole for 48 h, (d) PC3 and (e) DU145 cells were stained with Annexin V-FITC and PI and analyzed by flow cytometry.

Flow cytometry analysis was used to survey the impact of econazole on PC3 and DU145 cell cycle arrest. In Figure 8(a), we could observe that the percentages of PC3 cells at the G1 phase increased to 46.1%, 52.5%, 58.7%, and 60.5%, after the 48 h treatment with 5, 10, 20, and 40 *μ*M econazole, respectively, compared with that of the control group (G1 = 36.8%). For DU145 cells, treatment with the same concentration of econazole increased the percentages of cells in the G1 phase to 43.9%, 51.1%, 60.3%, and 65.9% for the respective econazole concentrations. The observations indicated that econazole could significantly increase the proportions of PC3 and DU145 cells in the G0/G1 phase in a dose-dependent manner. It was concluded that econazole arrested cells at the G0/G1 phase. To further study the mechanism of cell cycle arrest, western blot analysis was performed to detect the expression levels of key proteins at the G1 phase, including P-Rb, CyclinE, PUMA, and P53. As shown in Figure 8(b), econazole inhibited p-Rb and CyclinE expression and induced PUMA protein expression in a dose-dependent manner. The P53 protein expression level remained stable in PC3 cells but increasing in DU145 cells upon 5, 10, 20, and 40 *μ*M econazole treatment. Cell arrest-mediated P53 might be different in DU145 cells (bearing mutant p53) and PC3 cells (lacking p53). Econazole-induced cell arrest in DU145 was associated with alteration of p53 activation in DU145 cells via a dose-dependent manner. Actually, flow cytometry analysis also showed that the dose-dependent effect on cell arrest is more sensitive in DU145 cells than PC3 cells.

**Figure 9:**
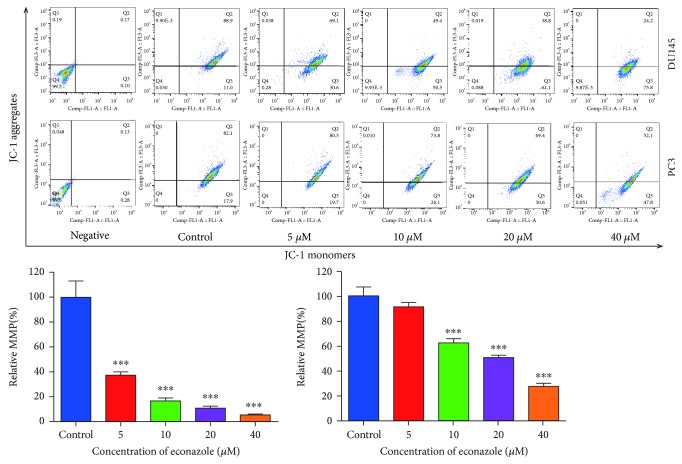
Econazole decreases mitochondrial membrane potential (MMP) in PCa cells. Relative MMP represents the MMP of the respective sample over that of control (%). The data was reported by mean ± SD from three experiments. ^∗^ *P* < 0.05, ^∗∗^ *P* < 0.01, and ^∗∗∗^ *P* < 0.001.

Insight into the effect of econazole on cell apoptosis was provided by flow cytometry analysis. Econazole obviously increased the apoptosis of PC3 and DU145 cells (Figures 8(d) and 8(e)). These consistent findings in the two cell types supported the idea that econazole induced apoptosis of PC3 and DU145 cells in a dose-dependent manner. We examined the expression of apoptosis-related proteins by western blot. Econazole reduced the expression of Bcl-2 and Mcl-1 and increased the expression of cleaved PARP in a dose-dependent manner (Figure 8(c)). Decreased MMP is an early manifestation of apoptosis and a hallmark of endogenous apoptosis. The ratio of mitochondrial depolarization was evaluated using the relative ratio of JC-1 polymer (red fluorescence) to JC-1 monomer (green fluorescence). As shown in Figure 9, the proportion of JC-1 monomers increased gradually and the relative ratio of JC-1 polymer to JC-1 monomer gradually decreased, which indicated that econazole can decrease the MMP levels of PC3 and DU145 cells. Therefore, econazole can induce cell apoptosis in PC3 and DU145 cells by activation of the endogenous apoptotic pathway but the exogenous apoptotic mechanism needs more investigations.

#### 3.6.2. Effect of Econazole on Cell Migration, Invasion, and Adhesion

**Figure 10:**
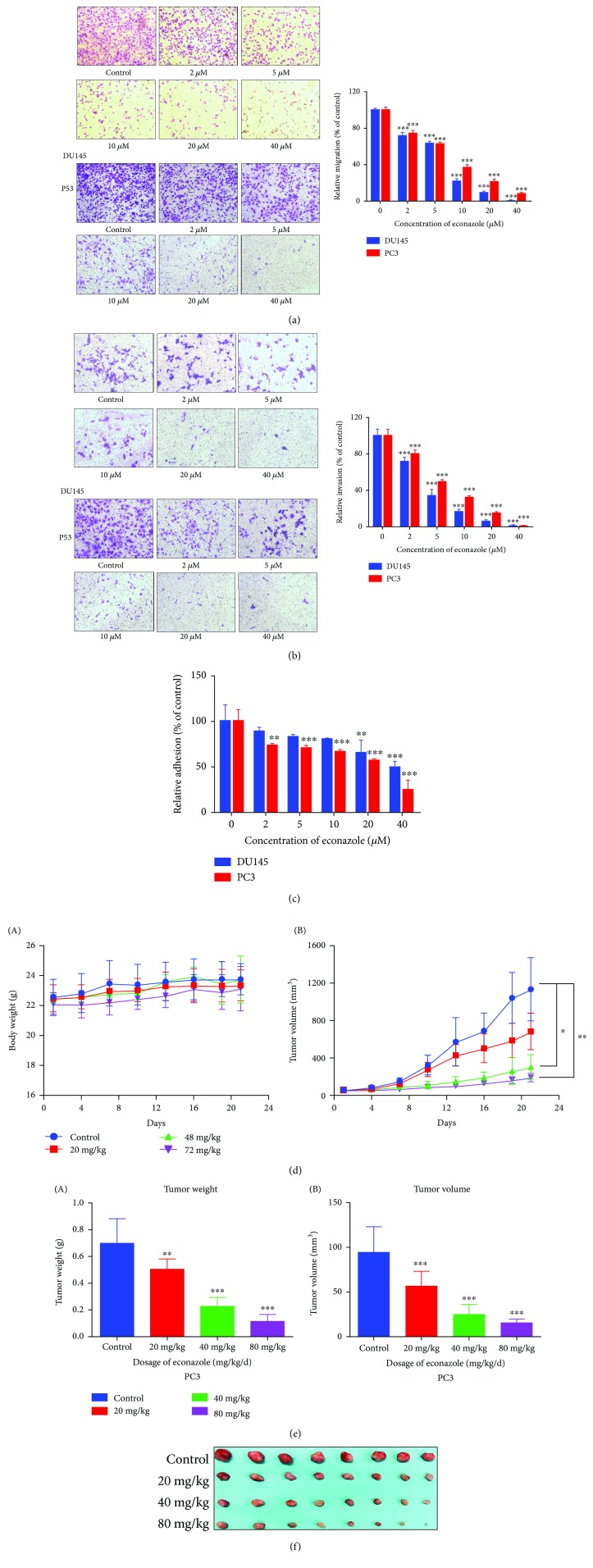
Effect of econazole on the migration, invasion, and cell adhesion and therapeutic effects on a nude mouse xenograft model. Transwell chambers were used to mimic the in vivo migration and invasion process. (a) The number of PCa cells that migrated to the membrane gradually decreased with increasing concentrations of econazole, especially 10, 20, and 40 *μ*M, and the relative mobility was <40% (original magnification, 100x). (b) The invasion of PC3 and DU145 cells was inhibited in a dose-dependent manner by econazole. (c) Effect of econazole on the cell adhesion of PCa cells. After treatment with different concentrations of econazole for 48 h, the number of PC3 and DU145 cells adhering to 96-well plates was significantly reduced in a dose-dependent manner. (d, e) The growth of PCa xenografts in nude mice inhibited by econazole. No differences in body weight was evident in the four groups. From days 5 to 21, tumor growth was rapid in the control group but was remarkably reduced in the econazole treatment groups for 40 and 80 mg/kg econazole. (f) Tumor size was also significantly reduced in the econazole-treated groups compared to the vehicle control. The data is reported as the mean ± standard deviation from three experiments. ^∗^ *P* < 0.05, ^∗∗^ *P* < 0.01, and ^∗∗∗^ *P* < 0.001.

To investigate the effect of econazole on the migration of PCa cells, we used Transwell chambers to mimic the *in vivo* migration process. The number of cells that migrated to the membrane gradually decreased with increasing concentrations of econazole, especially at 10, 20, and 40 *μ*M, and the relative mobility was <40% (Figure 10(a)).

Cell invasion is a process in which cells adhere to the extracellular matrix and secrete matrix metalloproteinases to degrade the extracellular matrix to complete migration, which is a key step in the metastasis of cancer cells. We also use the Transwell chambers as an invasion model. As the concentration of econazole increased, the amount of cell-derived Matrigel through the membrane decreased significantly (Figure 10(b)). Adhesion of adjacent cells or with the extracellular matrix during cell invasion and metastasis is an important process for tumor cells to enter the blood vessels and lymphatic vessels to achieve metastasis. Therefore, we studied the effect of econazole on PCa cell adhesion. After treatment with different concentrations of econazole for 48 h, the number of PC3 and DU145 cells adhering to 96-well plates was significantly reduced in a dose-dependent manner (Figure 10(c)). These results indicated that econazole significantly inhibited the migration, invasion, and adhesion abilities of PCa cells.

#### 3.6.3. Therapeutic Effects on the Xenograft Model

**Figure 11:**
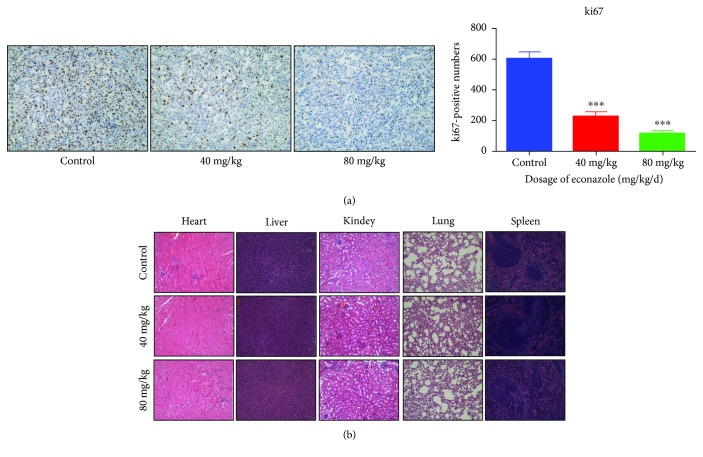
Econazole inhibited prostate tumor growth in the xenograft mouse model and had no toxic effect on the main organs (original magnification, 100x). (a) Ki67 immunohistochemical staining showed that the expression of Ki67 in the econazole treatment groups was significantly lower than that in the control group. (b) HE staining of paraffin-embedded sections of the heart, liver, kidney, lung, and spleen indicated that econazole inhibited the growth of tumors at 40 mg/kg and 80 mg/kg and also did not produce toxic side effects on nude mice.

Xenografts in the nude mouse model were used to investigate the therapeutic effects of econazole against PCa. BALB/c nude mice were subcutaneously implanted with PC3 cells. Intraperitoneal drug treatment with 20, 40, or 80 mg/kg econazole or vehicle control began the day after the first tumor cell injection. Animals were treated once a day, and tumor weight and volume were measured every 4 days. The study was terminated after 21 days. No difference in body weight was observed evidently in the four groups (Figure 10(d)). There was no significant difference in tumor growth between the groups in the first 4 days after tumor cell inoculation. However, from days 5 to 21, tumor growth was rapid in the control group but was remarkably reduced in the treatment groups, especially for 40 and 80 mg/kg econazole. Tumor size was also significantly reduced in the econazole-treated groups compared to the vehicle control (Figures 10(d)–10(f)). Ki67 is a proliferating cell-associated nuclear antigen whose function is closely related to mitosis and is indispensable in cell proliferation. The results of Ki67 immunohistochemical staining indicated that the expression of Ki67 in the econazole treatment groups was significantly lower than that of the control group, which further indicated that econazole inhibited the growth of prostate xenografts in nude mice (Figure 11(a)). Base on the hematoxylin-eosin (HE) staining result (Figure 11(b)), there was nontoxic effect on nude mice at the doses of 40 mg/kg and 80 mg/kg.

## 4. Discussion

Although PCa-specific lncRNAs are being increasingly identified, our understanding of lncRNAs for competing miRNA that regulates tumorigenesis and progression of PCa is still incomplete. The recently proposed ceRNA hypothesis posits that a novel form of posttranscriptional gene regulation operates through miRNA competition. With the identification of ceRNA crosstalk, miRNA, lncRNA, and their targeted genes can connect directly or indirectly. Herein, based on ceRNA hypothesis, we utilized paired miRNA, lncRNA, and mRNA expression profiles of PCa patients to construct a ceRNA network.

*PCA3* and *PCAT1* as well-known PCa-associated lncRNAs [4, 9] were identified in the ceRNA network. They were highly expressed with logFC values of 3.36 and 2.71, respectively. Interestingly, *UCA1* was downregulated in PCa compared with normal samples but was highly expressed in LNM samples and was also a main signature for machine learning model building. More importantly, *UCA1* was associated with the tumor stage with a *P* value of 0.04. Therefore, *UCA1* positively correlated with the severity of PCa. *UCA1* was originally discovered to be overexpressed in bladder cancer [48]. Several recent studies reported that *UCA1* might have a prognostic value in PCa and be a potential therapeutic target [49, 50]. Recently, it was reported that UCA1 could exacerbate oxidative stress and attenuates autophagy-dependent cell death through blocking autophagic flux [51, 52].

*XIST* is also a potential signature for the progression of PCa. *XIST* was significantly downregulated in both PCa and LNM samples, and the *XIST*/miR 372/DUSP2 axis might be important for ceRNA crosstalk according to the findings of a permutation test. *XIST* is required for X chromosome inactivation (XCI) and enables dosage compensation between XX females and XY males [53]. *XIST* loss may result in X reactivation and consequent genome-wide changes that lead to cancer, indicating that *XIST* RNA was required to maintain XCI and to suppress cancer *in vivo* [54]. *XIST* could engage in TGF-*β*-induced epithelial-mesenchymal transition (EMT) and cell invasion and metastasis in non-small-cell lung cancer (NSCLC) [55] and colorectal cancer (CRC) [56]. miR 372 is crucial in several human cancers [57]. Excessive miR 372 promotes metastasis of oral and liver cancers [58], but the role of *XIST* in PCa is rarely studied. We found that miR 372 was also upregulated in PCa metastasis samples, and *DUSP2*, the target gene of miR 372, is downregulated.

DUSP2 was also known as PAC1 (phosphatase of activated cells 1) and is a dual threonine/tyrosine phosphatase that specifically dephosphorylates and inactivates mitogen-activated protein (MAP) kinases. *DUSP2* transcription is induced in response to serum deprivation and oxidative stress, which leads to p53-dependent apoptosis [59]. DUSP2 also has been demonstrated to be involved in EMT through its direct involvement in the inactivation of the extracellular signal-regulated kinase pathway in pancreatic ductal adenocarcinoma, which is essential to the epithelium-originated solid tumor metastasis cascade [60]. Therefore, we hypothesize that *XIST* might regulate PCa progression and metastasis by competing for miR 372 to modulate the expression of DUSP2. The underlying mechanism remains unclear and should be further investigated.

Lymphatic metastasis is a common outcome of PCa and is one of the key factors affecting the prognosis of PCa patients. Thus, it is essential to discover biomarkers that accurately indicate the risk of lymphatic metastasis. We established four machine learning classifiers to discriminate LNM from non-LNM based on the signatures from the coexpression network. There were 32 significant signatures used to build the RF model. Among the signatures, *UCA1* and *XIST* were also the key hub nodes of the ceRNA network, suggesting that they might play important roles in the pathogenesis and metastasis of PCa. Some signatures have direct and indirect relationships with PCa or other types of cancer. For example, *CLDN2* is an X-linked oncogene or tumor suppressor gene in breast cancer [61]. *EMX1* and *EMX2* are homeodomain-containing transcription factors, and the function of *EMX2* has been linked to the WNT signaling pathway, which has an important role as a suppressor in lung cancer [62, 63]. Overexpression of *SCHLAP1* independently predicts the progression of lethal PCa [64]. Four signatures (*PATE1*, *PATE2*, *EDDM3A*, and *EDDM3B*) are extensively distributed in the prostate and testis. *UCA1*, *GABRG1*, *ANPEP*, *SCHLAP1*, and *CXCL5* were strongly related to the clinical stage of PCa. In particular, the *P* value of ANPEP of 1.096*e*-7 was consistent with a previous study [58]. Recently, it was reported that apoptosis-induced *CXCL5* accelerates inflammation and growth of prostate tumor metastases in the bone [65]. The relationship between *GABRG1* and cancer has not yet been reported. Presently, using enrichment analysis, we confirmed that the GABAergic system contributes to PCa progression [39]. It has also been reported that GABA is highly expressed in prostate tissue of patients with cancer or benign prostatic hypertrophy and the high expression has been observed in prostate tissue of cancer patients with LNM [66]. Therefore, the GABAergic system and its related dysregulated genes could be important in the progression and prognosis of cancer.

Another successful application of the coexpression network is to find drugs for PCa treatment utilizing the CMAP database. In this study, the anticancer effect of econazole was identified for further validation *in vitro* and *in vivo*.

CCK-8 and Edu staining assays showed that econazole significantly inhibited the proliferation of PC3 and DU145 cells. Flow cytometry and western blot were used to analyze apoptosis and cell cycle arrest in PC3 and DU145 cells after econazole treatment. Econazole effectively promoted apoptosis of PCa cells, including reduction of MMP, activation of caspase family proteins, cleavage of PARP-1, and decrease of Bcl-2 and Mcl-1 in a dose-dependent manner. Econazole also significantly regulated cell cycle arrest at the G0/G1 phase by inhibiting P-Rb and CyclinE protein expression and increasing PUMA and P53 protein expression. The antimetastasis activity of econazole was observed in Transwell chamber migration and human fibronectin assays. Econazole remarkably inhibited PCa cell migration, invasion, and adhesion. More importantly, econazole also inhibited the growth of prostate xenografts in nude mice. Thus, the result showed the anticancer effects of econazole and supported the novel therapeutic indication and usage of econazole for PCa treatment.

## 5. Conclusions

The PCa-associated dysregulated ceRNA network was developed by utilizing sample-matched miRNA, lncRNA, and mRNA expression profiles in combination with the miRNA regulatory network based on the ceRNA hypothesis. *XIST*/miR 372/*DUSP2* in the ceRNA network was speculated to participate in the EMT process. The underlying mechanism should be further investigated. In addition, the coexpression network was constructed to find the hub genes, which are selected as signatures for building the RF classifier to predict the PCa lymphatic metastasis. These key RNAs in the ceRNA network and lymphatic metastasis-associated signatures were analyzed by KS test. Taken together, we found that *UCA1*, *GABRG1*, *ANPEP*, *SCHLAP1*, and *CXCL5* were strongly related to the clinical stage of PCa and also had good performance for predicting lymphatic metastasis risk. Finally, based on the CMAP database, econazole was identified as a novel repositioning candidate for PCa treatment. *In vitro* and *in vivo* pharmacodynamic experiments validated that econazole could induce growth restraint, arrest cell cycle at the G0/1 phase, lead to apoptosis, inhibit migration, invasion, and adhesion in PCa cells, and inhibit the growth of prostate xenografts in nude mice. Although the exact molecular mechanism of the anticancer and antimetastasis activities of econazole remains unclear, the present findings indicate that econazole might be a potential therapeutic drug for PCa. Therefore, the study provides a substantial and feasible approach for identifying the potential diagnostic biomarker and therapeutic drug for the diagnosis and treatment of PCa.

## Figures and Tables

**Table 1:** Performance of classification models by 5-fold cross-validation, random sampling, and leave-one-out validation.

Method	5-fold cross-validation		Random sampling		Leave-one-out	
	AUC	Precise	AUC	Precise	AUC	Precise
Logistic regression (LR)	0.442	0.683	0.528	0.640	0.459	0.706
Random forest (RF)	0.840	0.842	0.815	0.844	0.775	0.840
*k* nearest neighbors (kNN)	0.581	0.712	0.648	0.753	0.571	0.719
Naive Bayesian (NB)	0.772	0.821	0.762	0.810	0.760	0.815

**Table 2:** Small molecules that potentially reverse dysregulation of expression profile in lymphatic metastasis and PCa samples, respectively.

CMAP drug name	Enrichment score	Mean score	*P* value
*Non-LNM compared with LNM samples*			
Geldanamycin	-0.983	-0.806	0.001
Econazole	-0.956	-0.804	0.002
6-Bromoindirubin-3′-oxime	-0.953	-0.616	0.005
Triamterene	-0.942	-0.449	0.007
Rilmenidine	-0.941	-0.341	0.007
Fludrocortisone	-0.918	-0.388	0.014
Mimosine	-0.909	-0.373	0.017
Tocainide	-0.896	-0.384	0.022
Ethoxyquin	-0.871	-0.330	0.033
Acenocoumarol	-0.812	-0.441	0.049
*Normal tissue compared with PCa tissue*			
Econazole	-0.972	-0.784	0.002
Rilmenidine	-0.968	-0.719	0.002
6-Bromoindirubin-3′-oxime	-0.878	-0.529	0.004
Diphenhydramine	-0.957	-0.446	0.004
Naftidrofuryl	-0.955	-0.336	0.004
PF-00539758-00	-0.952	-0.343	0.005
Meteneprost	-0.950	-0.408	0.005
Quipazine	-0.947	-0.397	0.006
Benzamil	-0.841	-0.467	0.008
Azacyclonol	-0.933	-0.368	0.009

## Data Availability

The expression profile data supporting this study were retrieved from The Cancer Genome Atlas (TCGA) data portal (version October, 2017), which have been mentioned in the manuscript. The processed data are available in the supplementary files, and more information can be obtained from the corresponding authors upon request.

## Supplementary Materials

Supplementary Materials Figure S1: the prognostic value of lncRNAs in PCa was evaluated by KS test, by computing expression changes of lncRNAs with the progression of PCa among stages II, III, and IV. *P* values of UCA1 (A) and OSTN-AS1 (B) were <0.05. UCA1 was downregulated in PCa tissue, but its expression gradually increased with the progression of PCa, and UCA1 was upregulated in PCa lymphatic metastasis. SCHLAP1 (C), LINC01141 (D), CTD-2521M24.5 (E), and RP11-245J24.1 (F) were also identified as important progression of PCa-related lncRNAs with *P* values < 0.01. Figure S2: proliferation inhibitory effect of dose response curves of geldanamycin and 6-bromoindirubin-3′-oxime (BIO). Geldanamycin on prostate cancer cells, PC3 cells (A), and DU145 cells (C) was cultured in 96-well plates with different concentrations of econazole for 72 h. Dose response curves of BIO on PC3 and DU145 cells were shown in Figure S1 B and D, respectively. Cell viability was measured by CCK-8 assay. Each bar represents the mean ± SD of three determinations.
