# Supplementary material for: Competing Endogenous RNA and Coexpression Network Analysis for Identification of Potential Biomarkers and Therapeutics in association with Metastasis Risk and Progression of Prostate Cancer
Source: Oxid Med Cell Longev. 2019 Aug 5;2019:8265958. doi: 10.1155/2019/8265958 (PMC6701351; doi:10.1155/2019/8265958)
Supplement: Supplementary Materials — Figure S1: the prognostic value of lncRNAs in PCa was evaluated by KS test, by computing expression changes of lncRNAs with the progression of PCa among stages II, III, and IV. P values of UCA1 (A) and OSTN-AS1 (B) were <0.05. UCA1 was downregulated in PCa tissue, but its expression gradually increased with the progression of PCa, and UCA1 was upregulated in PCa lymphatic metastasis. SCHLAP1 (C), LINC01141 (D), CTD-2521M24.5 (E), and RP11-245J24.1 (F) were also identified as important progression of PCa-related lncRNAs with P values < 0.01. Figure S2: proliferation inhibitory effect of dose response curves of geldanamycin and 6-bromoindirubin-3′-oxime (BIO). Geldanamycin on prostate cancer cells, PC3 cells (A), and DU145 cells (C) was cultured in 96-well plates with different concentrations of econazole for 72 h. Dose response curves of BIO on PC3 and DU145 cells were shown in Figure S1 B and D, respectively. Cell viability was measured by CCK-8 assay. Each bar represents the mean ± SD of three determinations. [file 8265958.f1.pdf]

Supplement figure:

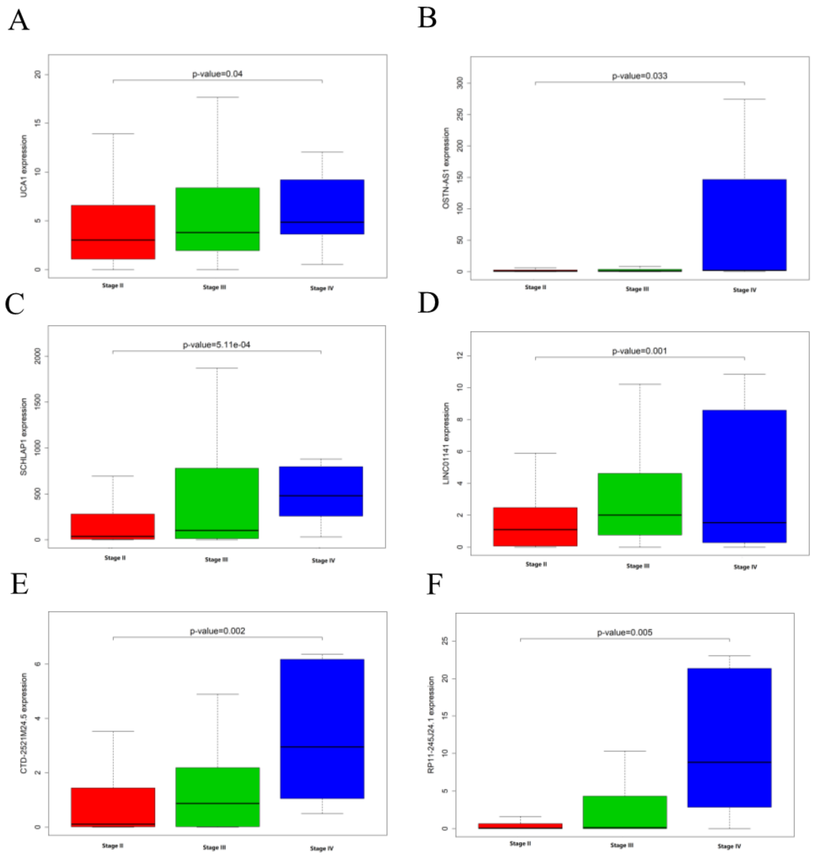

Figure S1. The prognostic value of lncRNAs in PCa were evaluated by KS test, for by computing expression changes of lncRNAs with the progression of PCa among stages II, III, and IV.P-value of *UCA1* (A) and *OSTN-AS1* (B) were <0.05. UCA1 was down-regulated in PCa tissue, but its expression gradually increased with the progression of PCa nad UCA1 was up-regulated in PCa lymphatic metastasis. *SCHLAP1*(C), *LINC01141*(D), *CTD-2521M24.5*(E), and *RP11-245J24.1* (F) were also identified as important progression of PCa-related lncRNAs with P-values <0.01.

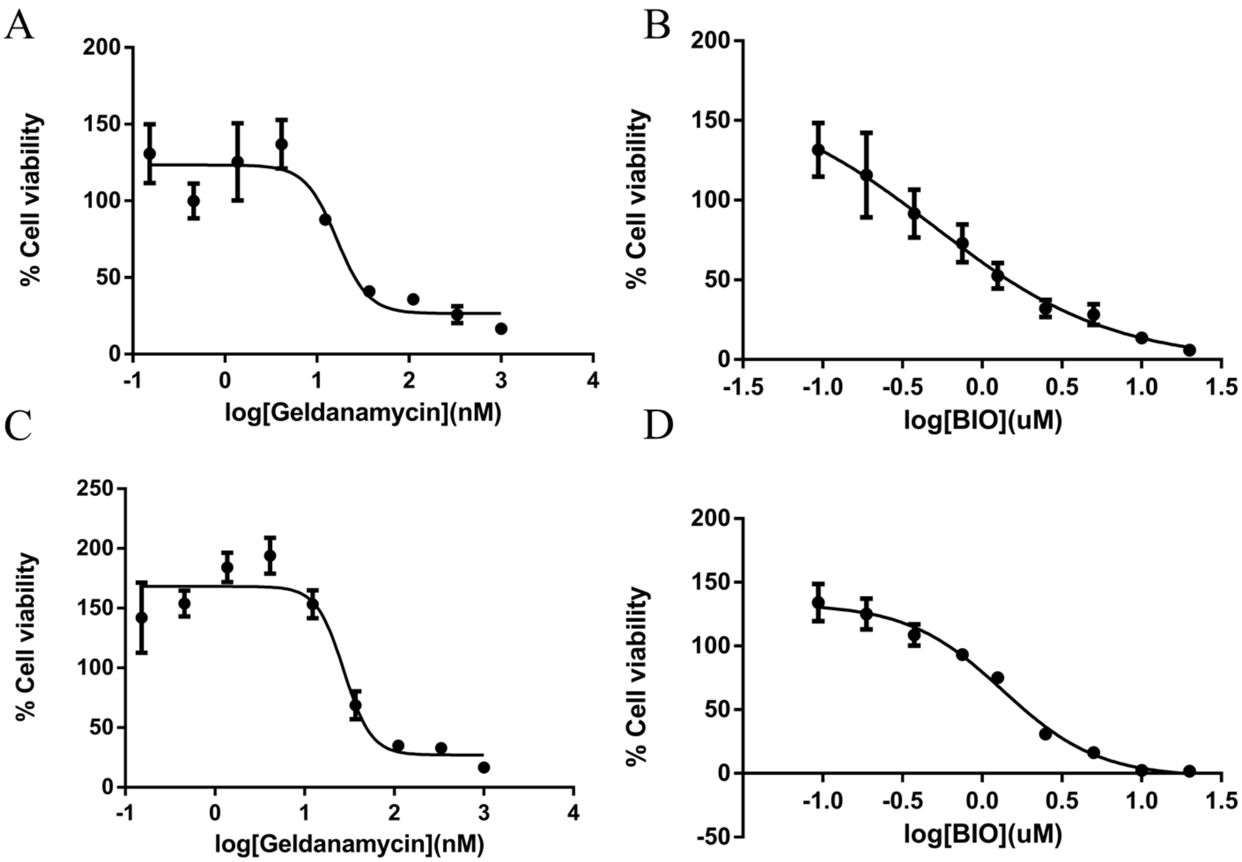

Figure S2: Proliferation inhibitory effect of dose response curves of geldanamycin and 6-bromoindirubin-3'-oxime (BIO). Geldanamycin on prostate cancer cells, PC3 (A) and DU145(C) cells were cultured in 96-well plates different concentration of econazole for 72h. Dose response curves of BIO on PC3 and DU145 cells were shown in figureS1 B and D, respecitively. Cell viability was measured by CCK-8 assay. Each bar represents the mean± SD of three determinations.
